# hsa_circ_0023305 Enhances Laryngeal Squamous Cell Carcinoma Progression and Modulates TRPM7 via miR-218-5p Sponging

**DOI:** 10.1155/2021/9968499

**Published:** 2021-12-02

**Authors:** Yi Zhang, Kaisai Tian, Enhui Zhou, Xiaocheng Xue, Shiling Yan, Yixin Chen, Peipei Qiao, Liyun Yang, Xiaoping Chen

**Affiliations:** ^1^Department of Otorhinolaryngology Head and Neck Surgery, Shanghai Pudong New Area Gongli Hospital, Shanghai 200135, China; ^2^Ningxia Medical University, Yinchuan, Ningxia 750004, China

## Abstract

Recently, circular RNAs have been shown to function as critical regulators of many human cancers. However, the circRNA mechanism in laryngeal squamous cell carcinoma (LSCC) remains elusive. Recent investigations using bioinformatics analysis revealed high expression of hsa_circ_0023305 in LSCC tissues compared to normal tissues. Furthermore, we discovered that hsa_circ_0023305 expression level was positively correlated to tumor/node/metastasis (TNM) stage as well as lymph node metastasis in LSCC. Moreover, higher hsa_circ_0023305 levels were correlated to poorer LSCC patient outcomes. Knockdown of hsa_circ_0023305 significantly inhibited LSCC cell proliferation, invasion, and migration abilities. Our team validated that hsa_circ_0023305 functioned as a miR-218-5p sponge from a mechanistic perspective, targeting the melastatin-related transient receptor potential 7 (TRPM7) in LSCC cells. TRPM7 regulates a nonselective cation channel and promotes cancer proliferation and metastasis. Our data demonstrated that miR-218-5p was downregulated in LSCC and that miR-218-5p upregulation repressed LSCC proliferation and invasion both *in vivo* and *in vitro*. Additionally, we found that hsa_circ_0023305-mediated upregulation of TRPM7 inhibited miR-218-5p and contributed to LSCC migration, proliferation, and invasion. In summary, these data propose a new mechanism by which the hsa_circ_0023305/miR-218-5p/TRPM7 network enhances LSCC progression.

## 1. Introduction

Laryngeal cancer is a malignancy of the larynx that accounts for 1–5% of total malignant tumor incidence. Laryngeal squamous cell carcinoma (LSCC) is the most general class of laryngeal cancer [1]. Currently, LSCC treatment primarily includes radiation therapy, surgery, and chemotherapy along with biological therapy [2]. The five-year survival rate of LSCC is greater than 90% with early treatment; metastasis and recurrence are the main factors that influence LSCC prognosis. Poor differentiation and lymph node metastases are associated with reduced five-year survival rate [3]. As such, early diagnosis and treatment are extremely indispensable for lowering laryngeal cancer mortality.

Recently, circular RNAs (circRNAs) have received attention regarding their correlation with human diseases, particularly cancer, suggesting a potential use for circRNAs as novel biomarkers and therapeutic targets [4, 5]. circRNAs are an abundant type of endogenous RNA and have recently been rediscovered and reevaluated for their essential functions in the regulation of gene expression [5]. circRNAs primarily function as noncoding RNA molecules that are comprised of a covalently closed continuous loop without 5′-3′ polarity or a poly-A tail. circRNAs have a relatively jarless framework, and their expression is tissue-specific in the eukaryotic transcriptome. Emerging evidence has identified the presence of endogenous circRNAs in mammalian cells [6]. These circRNAs regulate gene expression at the posttranscriptional or transcriptional level by binding to and inhibiting the function of microRNAs (miRNAs) and other molecules [6]. Previous studies have found that circRNA_100290 overexpression enhances LSCC progression via the miR-136-5p/RAP2C axis [7]. The circular RNA circCORO1C promotes LSCC progression via modulation of the let-7c-5p/PBX3 axis [8].

Our study with high-throughput sequencing identified many circRNAs with abnormal expression. Among these circRNAs, hsa_circ_0023305 expression was increased in LSCC tissues. However, the precise relationship between hsa_circ_0023305 expression and LSCC progression remains unclear. Therefore, the current study is aimed at revealing the regulatory mechanism concerning hsa_circ_0023305 expression in LSCC.

## 2. Materials and Methods

### 2.1. Tissue Samples

In total, we obtained 30 paired LSCC tissues along with adjacent nontumor tissues from patients in the hospital. No patients received chemotherapy and/or radiotherapy prior to surgery. We obtained written informed consent from all patients. The Ethics Committee in Shanghai Gongli Hospital approved the investigation.

### 2.2. Cell Culture and Transfection

We received AMC-HN-8 and Hep-2 cells from Cell Bank of Chinese Academy of Sciences (Shanghai, China). We maintained cells in Dulbecco's modified Eagle medium (DMEM, Gibco, CA, USA) with 10% fetal bovine serum (FBS, Gibco) at 37°C in a humidified atmosphere with 5% CO_2_.

We obtained hsa_circ_0023305 interference lentiviral vectors (sh-hsa_circ_0023305: forward 5′-CCCGCACGAUUUCAUUGAAUU-3′ and reverse 5′-UUGGGCGUGCUAAAGUAACUU-3′), melastatin-related transient receptor potential 7 (TRPM7) overexpression vector (cDNA of TRPM7 sequence was cloned into the cDNA3.1 vector), miR-218-5p inhibitors (5′-CCAUGUGGUUGCGAGGUAUGA-3′), and negative controls from GenePharma (Shanghai, China). We performed cell transfection using Lipofectamine 2000 reagents (Invitrogen, Carlsbad, CA, USA), according to the manufacturer's instructions. Cells were used for experimentation after culturing for another 48 h after transfection.

### 2.3. Fluorescence *In Situ* Hybridization (FISH)

The hsa_circ_0023305-specific FITC-labeled probes were used to perform fluorescence *in situ* hybridization (FISH). Nuclei with 4,6-diamidino-2-phenylindole (DAPI) were counterstained and experiments were conducted according to kit protocols (GenePharma, Shanghai, China). The hsa_circ_0023305 probe sequence was 5′-ACTGGTAAAACCCCAGTCTGCGAGGAACAGAAG-3′.

### 2.4. Bioinformatics Analysis

We predicted interactive correlations between circRNA, miRNA, and mRNA based upon http://starbase.sysu.edu.cn/.

### 2.5. Quantitative Real-Time Polymerase Chain Reaction (qPCR)

We obtained RNA from cells or from skin wound tissue using the TRIzol reagent kit (Invitrogen, Carlsbad, CA, USA). We synthesized and amplified cDNA using a TaqMan miRNA Reverse Transcription Kit. We conducted qPCR using the TaqMan™ MicroRNA Assay Kit (Applied Biosystems, Foster City, CA, USA), and we employed the 2 − ΔΔCT method to detect relative expression fold changes. The thermal cycle was as follows: predenaturation at 95°C for 1 min, followed by 40 cycles at 95°C for 15 s, 60°C for 30 s, and 72°C for 30 s. Our team utilized *U6* and *GAPDH* as internal references. We used the following primers to assay hsa_circ_0023305 expression: forward 5′-GCAGAAGACAAAAAGACAAAC-3′ and reverse 5′-GGTCCAGGTAGTTCATGGCCAGC-3′. The miR-218-5p primers were as follows: forward 5′-AACACGAACTAGATTGGTACA-3′ and reverse 5′-AGTCTCAGGGTCCGAGGTATTC-3′. TRPM7 primers were as follows: forward 5′-CTTTGACCAAGAGGGAATGTG-3′ and reverse 5′-GACCAAGCGACCACAAAAAC-3′. U6 primers included the following: forward 5′-CTCGCTTCGGCAGCACA-3′ and reverse 5′-AACGCTTCACGAATTTGCGT-3′. GAPDH primers were as follows: forward 5′-AATGGGCAGCCGTTAGGAAA-3′ and reverse 5′-TGAAGGGGTCATTGATGGCA-3′.

### 2.6. Cell Counting Kit-8 (CCK-8) Assay

Two days after transfection, we collected cells and plated them in 96-well plates at 2,000 cells/well. After culturing for 0, 1, 2, or 3 d, we added 10 *μ*L CCK-8 solution (Gibco, Logan, Utah, USA) per well and incubated for 1 h. Then, we measured the optical density (OD) value of each well using a microplate reader with 490 nm wavelength.

### 2.7. Colony Formation Assay

Cells were collected 48 h after transfection, seeded at a density of 200 cells/well in a 6-well plate, and cultured with complete medium for 10 d. We fixed cell colonies with 4% paraformaldehyde for 15 minutes and stained them with 0.1% crystal violet for 5 minutes. We photographed and counted colonies in a light selective environment.

### 2.8. Transwell Assay

Transfected cells were incubated with serum-free DMEM for 0.5 d prior to the Transwell assay. We stored Matrigel (Corning Life Sciences, Corning, NY, U.S.A.) at 4°C overnight for experiments. We placed Transwell chambers into each well of a 24-well plate and added DMEM (500 *μ*L; containing 20% FBS) to the lower chambers. We also added 1 × 10^5^ transfected cells in 100 *μ*L serum-free DMEM to the upper chambers to assess migration ability. Alternatively, we added cells into upper chambers coated with 10 *μ*L Matrigel to assess invasion. After 2 d of conventional incubation, we removed the Transwell chambers. We lightly wiped the inner cells away and placed chambers in a new 24-well plate with 4% paraformaldehyde (600 *μ*L). Cells were fixed for 5 minutes and then stained with 0.1% crystal violet for 10 minutes. Lastly, a technician counted cells in five random fields using an inverted microscope.

### 2.9. Western Blots

Protein was extracted from cells or tissues with RIPA lysis buffer, and Western blots were completed. Primary antibodies were against TRPM7 and GAPDH (Cell Signaling Technology, Beverly, MA, USA), and we stained blots according to the suggested protocol. Immunoreactivity was visualized with chemiluminescence detection kits (Western Blotting Substrate, Donghuan Biotech, China).

### 2.10. Dual-Luciferase Reporter Assay

The putative miR-218-5p binding site in the 3′-UTR of hsa_circ_0023305 [wild-type (WT) or mutant (MUT)] and target gene TRPM7 were cloned into the psi-CHECK vector (Promega, Madison, Wisconsin, USA). The binding sites were cloned in downstream of the firefly luciferase 3′-UTR or hsa_circ_0023305 as a primary luciferase signal. Renilla luciferase was used as the normalization signal. Constructs were labeled TRPM7-Wt/hsa_circ_0023305-Wt and TRPM7-Mut/hsa_circ_0023305-Mut. The psi-CHECK vector supplied the Renilla luciferase normalization signal to compensate for alterations resulting from transfection and harvesting efficiencies. Transfection into HEK293 cells was achieved using Lipofectamine 2000 (Invitrogen Life Technologies, Carlsbad, USA). We detected Renilla and firefly luciferase activities 1 d after transfection using the Dual-Luciferase Reporter Assay System (Promega, Mannheim, Germany) and a luminometer (Molecular Devices, U.S.A.). Relative Renilla luciferase activities were detected according to the manufacturer's instructions (Promega, Mannheim, Germany).

### 2.11. Animal Studies

For xenograft assays, mice were subcutaneously injected with 1 × 10^6^ AMC-HN-8 cells after treatment with sh-NC or sh-hsa_circ_0023305. We measured LSCC tumor volume (length × width^2^ × 0.5) every 5 d and LSCC tumor weight following 30 days of injections.

To assess metastasis, 2 × 10^5^ sh-NC or sh-hsa_circ_0023305 AMC-HN-8 cells with luciferase expression vectors were injected into mouse tails intravenously. After 1 month, AMC-HN-8 cell metastases were analyzed through bioluminescence imaging, following intravenous tail luciferin injection (150 mg luciferin/kg body weight).

### 2.12. Statistical Analysis

Data are reported as the mean ± standard deviation (SD). A statistician utilized GraphPad Prism (GraphPad, La Jolla, USA) to detect differences between groups. *P* values ≤ 0.05 were considered to be statistically significant.

## 3. Results

### 3.1. High Expression of hsa_circ_0023305 Correlates to Poor LSCC Prognosis

**Figure 1:**
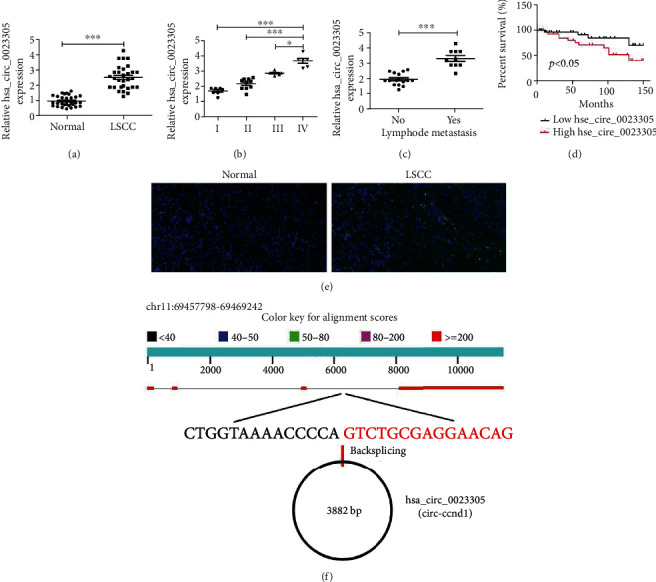
hsa_circ_0023305 expression is increased in LSCC. (a) RT-qPCR analysis for hsa_circ_0023305 expression in 30 paired LSCC tissues. Data are presented as the mean ± SD; ^∗∗∗^*P* < 0.001. High hsa_circ_0023305 expression was associated with advanced tumor stage (b) and lymph node metastasis (c). Data are presented as the mean ± SD; ^∗^*P* < 0.05,  ^∗∗^*P* < 0.01, and^∗∗∗^*P* < 0.001. (d) High hsa_circ_0023305 expression was correlated with poor overall survival (OS) in patients with LSCC. (e) Fluorescence in situ hybridization (FISH) of hsa_circ_0023305 (green) combined with nuclear DAPI staining (blue) in LSCC tissues and adjacent tissue. (f) Location of hsa_circ_0023305 on the human chromosome.

RT-qPCR detection found that hsa_circ_0023305 expression was increased in LSCC tissues compared to adjacent tissue (Figure 1(a)). Higher hsa_circ_0023305 expression was also correlated to more advanced tumor stage (Figure 1(b), Table 1) as well as lymph node metastasis (Figure 1(c), Table 1). To detect the interplay between hsa_circ_0023305 and prognosis in laryngeal cancer patients, we plotted Kaplan-Meier survival curves. High hsa_circ_0023305 expression may be associated with the poor prognosis of laryngeal carcinoma. Specifically, higher hsa_circ_0023305 levels correlated with worse prognoses (Figure 1(d)). Fluorescence in situ hybridization (FISH) analysis demonstrated that hsa_circ_0023305 expression was increased in LSCC tissues compared to adjacent tissue and hsa_circ_0023305 was primarily located in cytoplasm (Figure 1(e)). Bioinformatics analysis found that hsa_circ_0023305 was comprised of four exons from the CCND1 gene. The mature hsa_circ_0023305 was located in chr11:69457798-69469242, and the spliced length was 3882 bp (Figure 1(f)).

### 3.2. hsa_circ_0023305 Downregulation Suppresses LSCC Cell Proliferation and Growth

**Figure 2:**
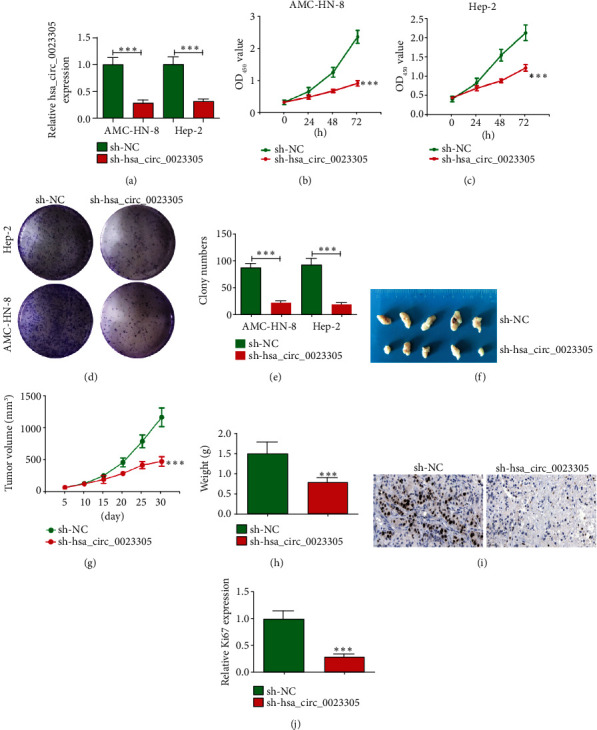
Downregulation of hsa_circ_0023305 suppresses LSCC cell proliferation and tumor growth. (a) hsa_circ_0023305 expression in Hep-2 and AMC-HN-8 cells transfected with sh-hsa_circ_0023305 or sh-NC. Data are presented as the mean ± SD; ^∗∗∗^*P* < 0.001. (b, c) CCK8 analysis shows the proliferative capacity of both Hep-2 and AMC-HN-8 cells after transfection with sh-hsa_circ_0023305 or sh-NC. Data are presented as the mean ± SD; ^∗∗∗^*P* < 0.001 vs. sh-NC. (d, e) Plate cloning experiments were performed to detect the number of LSCC cells transfected with sh-hsa_circ_0023305 or sh-NC in both Hep-2 and AMC-HN-8 cells. Data are presented as the mean ± SD; ^∗∗∗^*P* < 0.001. (f) AMC-HN-8 cells were transfected with sh-hsa_circ_0023305 or sh-NC and subcutaneously injected into nude mice. (g, h) Tumor growth curves and tumor weight were analyzed. Data are presented as the mean ± SD; ^∗∗∗^*P* < 0.001 vs. sh-NC. (i, j) Immunohistochemistry for the percentage of Ki67-positive cells. The relative Ki67 expression was calculated. Data are presented as the mean ± SD; ^∗∗∗^*P* < 0.001 vs. sh-NC.

To better analyze the regulatory effect of hsa_circ_0023305 on LSCC cell proliferation and tumor growth, we constructed a lentiviral vector with hsa_circ_0023305 interference stability. RT-qPCR analyses showed that hsa_circ_0023305 expression was significantly decreased in Hep-2 and AMC-HN-8 cells (Figure 2(a)). CCK8 analysis showed that silencing of hsa_circ_0023305 inhibited LSCC cell proliferation in both AMC-HN-8 and Hep-2 cells (Figures 2(b) and 2(c)). Plate cloning experiments were carried out to explore proliferative capacity in AMC-HN-8 and Hep-2 cell lines. Knockdown of hsa_circ_0023305 attenuated the proliferative capacity of AMC-HN-8 and Hep-2 cells compared to the sh-NC group (Figures 2(d) and 2(e)).

We also explored the impact of hsa_circ_0023305 expression on tumor growth *in vivo* using AMC-HN-8 cells and discovered that tumor volume (Figures 2(f) and 2(g)) and weight (Figure 2(h)) were clearly decreased in the sh-hsa_circ_0023305 group compared to the sh-NC group. Immunohistochemical detection showed that Ki67 expression was decreased in the sh-hsa_circ_0023305 group compared to the sh-NC group (Figures 2(i) and 2(j)). Together, our data suggest that hsa_circ_0023305 downregulation suppresses LSCC cell proliferation and tumor growth.

### 3.3. hsa_circ_0023305 Downregulation Decreases LSCC Cell Invasion *In Vivo* and *In Vitro*

**Figure 3:**
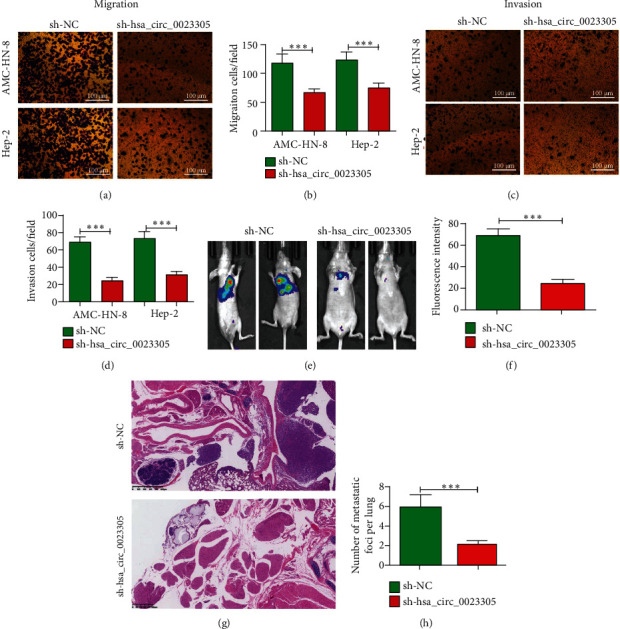
Downregulation of hsa_circ_0023305 decreases LSCC cell invasion in both *in vitro* and *in vivo* experiments. (a–d) Cell migration and invasion were analyzed in both Hep-2 and AMC-HN-8 cells using Transwell assays. Data are presented as the mean ± SD; ^∗∗∗^*P* < 0.001 vs. sh-NC.(e, f) Live imaging showing the effects of circ-TRPS1 on metastasis of AMC-HN-8 cells 30 d after intravenous tail injection. The relative fluorescence density was calculated. Data are presented as the mean ± SD; ^∗∗∗^*P* < 0.001. (g, h) The numbers of metastatic foci in lung tissues were calculated according to the HE staining. The data are expressed as the mean ± SD. ^∗∗∗^*P* < 0.001 vs. sh-NC.

Transwell invasion and migration studies found that silencing of hsa_circ_0023305 decreased LSCC cell invasion and migration in Hep-2 and AMC-HN-8 cells (Figures 3(a)–3(d)). Whole body imaging in mice illustrated that hsa_circ_0023305 knockdown inhibited tumor metastasis and development (Figures 3(e) and 3(f)). Living imaging detection shows the pulmonary metastasis and found that hsa_circ_0023305 silence decreased the pulmonary metastasis ability by decreasing the numbers of metastatic foci in lung tissues after HE staining analysis (Figures 3(g) and 3(h)). These data suggest that downregulation of hsa_circ_0023305 decreases LSCC cell invasion *in vitro* and *in vivo*.

### 3.4. miR-218-5p and TRPM7 Are hsa_circ_0023305 Downstream Targets

**Figure 4:**
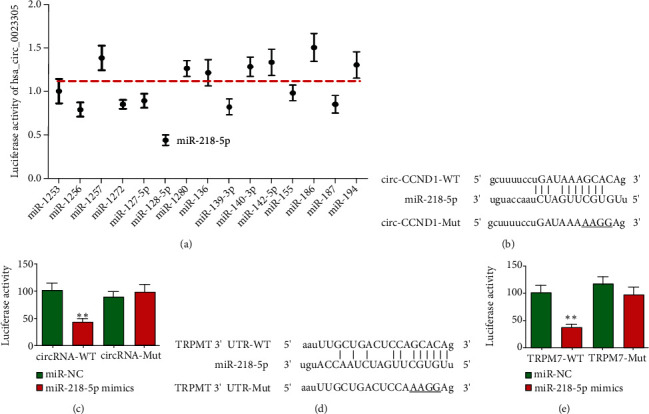
The miR-218-5p and TRPM7 are downstream targets of hsa_circ_0023305. (a) The luciferase activity of hsa_circ_0023305 in HEK293T cells transfected with different miRNA mimics, which are putative binding sites for the hsa_circ_0023305 sequence. Luciferase activity was normalized by Renilla luciferase activity. (b) Prediction of binding sites of miR-218-5p in hsa_circ_0023305. The MUT version of hsa_circ_0023305 is presented. (c) Relative luciferase activity was determined 48 h after transfection of HEK293T cells with miR-218-5p mimic/NC or hsa_circ_0023305 WT/Mut. Data are presented as the mean ± SD. ^∗∗^*P* < 0.01. (d) Prediction of binding sites of miR-218-5p within the 3′-UTR of TRPM7. The MUT version of 3′-UTR-TRPM7 is shown. (e) Relative luciferase activity was determined 48 h after transfection of HEK293T cells with miR-218-5p mimic/NC or 3′-UTR-TRPM7 WT/Mut. Data are presented as the mean ± SD. ^∗∗^*P* < 0.01.

Bioinformatics analyses demonstrated that hsa_circ_0023305 interacts with several miRNAs including miR-218-5p, miR-1256, miR-1257, miR-127-5p, miR-136, and miR-139-3p. We constructed different luciferase reporter constructs with inserted hsa_circ_0023305 sequences. After transfecting with different miRNA mimics, we discovered that only miR-218-5p mimics suppressed luciferase activity (Figure 4(a)). This suggested that miR-218-5p was a downstream target of hsa_circ_0023305. We next constructed WT and MUT hsa_circ_0023305 luciferase reporter plasmids (Figure 4(b)). The data suggest that luciferase activity decreased after cotransfection with WT hsa_circ_0023305 and miR-218-5p mimics (Figure 4(c)), suggesting that hsa_circ_0023305 interacted directly with miR-218-5p.

Bioinformatics analysis characterized TRPM7 as a miR-218-5p downstream target (Figure 4(d)). Luciferase reporter assay results validated that miR-218-5p was able to bind to the TRPM7 3′-UTR (Figure 4(e)).

### 3.5. Overexpression of TRPM7 or Inhibition of miR-218-5p Reverses the Effects of hsa_circ_0023305 Silencing on LSCC Cell Invasion and Proliferation

**Figure 5:**
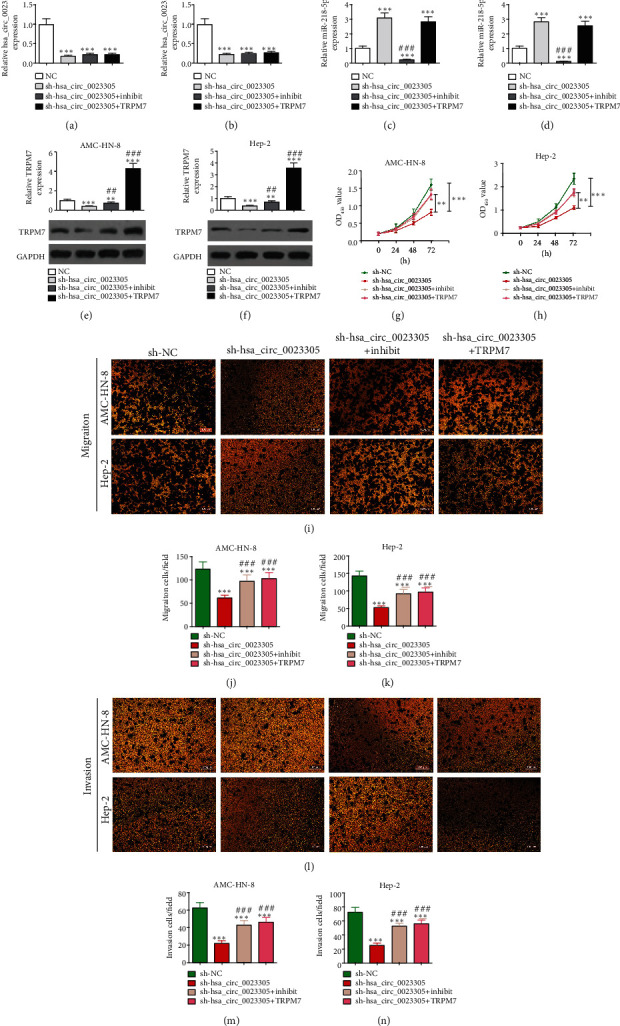
Overexpression of TRPM7 or inhibition of miR-218-5p reverses the effect of hsa_circ_0023305 silencing on LSCC cell proliferation and invasion. RT-qPCR detection showing the expression of hsa_circ_0023305 (a, b), miR-218-5p (c, d) in Hep-2, and AMC-HN-8 cells. Data are presented as the mean ± SD; ^∗∗∗^*P* < 0.001 vs. sh-NC; ^###^*P* < 0.001 vs. sh-hsa_circ_0023305. (e, f) RT-qPCR and Western blot detection showing the expression of TRPM7 in Hep-2 and AMC-HN-8 cells. Data are presented as the mean ± SD; ^∗∗^*P* < 0.01 and ^∗∗∗^*P* < 0.001 vs. sh-NC; ^##^*P* < 0.01 and ^###^*P* < 0.001 vs. sh-hsa_circ_0023305. (g, h) CCK8 analysis showed the proliferative ability of Hep-2 and AMC-HN-8 cells. Data are expressed as the mean ± SD. ^∗∗^*P* < 0.01 and ^∗∗∗^*P* < 0.001. Transwell detection showed the migration (i–k) and invasion (l–n) capacities of Hep-2 and AMC-HN-8 cells. Data are presented as the mean ± SD; ^∗∗∗^*P* < 0.001 vs. sh-NC; ^###^*P* < 0.001 vs. sh-hsa_circ_0023305.

We transfected AMC-HN-8 and Hep-2 cells with sh-hsa_circ_0023305, a miR-218-5p inhibitor, or a TRPM7-overexpression plasmid either in combination or alone. The RT-qPCR results demonstrated that hsa_circ_0023305 was successfully silenced and that hsa_circ_0023305 knockdown decreased miR-218-5p expression. TRPM7 overexpression did not recover hsa_circ_0023305 expression in either Hep-2 or AMC-HN-8 cells (Figures 5(a) and 5(b)), indicating that miR-218-5p and TRPM7 were downstream targets of hsa_circ_0023305. Furthermore, hsa_circ_0023305 knockdown increased miR-218-5p expression. TRPM7 overexpression did not downregulate miR-218-5p expression, even after hsa_circ_0023305 knockdown. Likewise, TRPM7 overexpression did not downregulate miR-218-5p even after hsa_circ_0023305 knockdown (Figures 5(c) and 5(d)), indicating that TRPM7 was a miR-218-5p downstream target. hsa_circ_0023305 knockdown decreased TRPM7 expression; however, decreased miR-218-5p expression partially rescued TRPM7 expression (Figures 5(e) and 5(f)), which confirmed that TRPM7 is a downstream target of miR-218-5p.

CCK8 analysis demonstrated that overexpression of TRPM7 or miR-218-5p inhibition reversed the effects of hsa_circ_0023305 silencing on LSCC cell proliferation in both AMC-HN-8 and Hep-2 cells (Figures 5(g) and 5(h)). Transwell migration and invasion assays illustrated that TRPM7 overexpression or miR-218-5p inhibition reversed the effects of hsa_circ_0023305 silencing on LSCC cell invasion as well as migration in Hep-2 and AMC-HN-8 cells (Figure 5(i)–5(n)).

## 4. Discussion

circRNAs are identified by their covalently closed loop structures that lack a 5′ cap and 3′ poly-A tail. circRNAs are broadly expressed in eukaryotic cells and possess important gene regulatory potential, including regulation of tumor formation and aggressiveness [8]. Previous studies have shown circRNAs participate in the development of resistance to treatment by modulating regulatory pathways and cellular processes, including the mitogen-activated protein kinase pathway, epithelial-mesenchymal transition, apoptosis, and autophagy [9]. The study also found that circRNA regulates gene expression through various mechanisms, such as acting as an “miRNA sponge,” regulating transcription and splicing, interacting with RNA-binding proteins, and even translating polypeptides [10]. Our study showed that hsa_circ_0023305 expression was increased in LSCC patients and higher hsa_circ_0023305 expression correlated to poorer prognosis, increased tumor stage, and more lymph node metastasis among LSCC patients. Additionally, higher hsa_circ_0023305 expression was associated with poorer overall survival (OS) of LSCC patients, suggesting that hsa_circ_0023305 plays an important role in LSCC. Bioinformatics analysis (https://starbase.sysu.edu.cn/) also found that hsa_circ_0023305 can interact with RNA-binding proteins including ELAVL1, U2AF2, TAF15, RBFOX2, and IGF2BP2. But the mechanism of circRNAs to interact with proteins, thereby binding, sequestering, or translocating proteins to particular subcellular fractions, allows circRNAs to act as dynamic scaffolds that modulate protein-protein interaction still largely unclear.

Downregulation of hsa_circ_0023305 suppressed LSCC cell proliferation and invasion both *in vitro* and *in vivo*. Bioinformatics results identified miR-218-5p as a hsa_circ_0023305 downstream target. Luciferase reporter assays validated the relationship between hsa_circ_0023305 and miR-218-5p. Previous studies have reported abnormal miR-218-5p expression in some diseases, including bladder cancer [11], non-small-cell lung cancer [12], gastric cancer [13], and pancreatic cancer [14]. Upregulation of miR-218-5p suppressed cancer proliferation and invasion [15]. The study also suggested that miR-218-5p was downregulated in invasive front cells and that this miRNA negatively regulated oral squamous cell carcinoma invasiveness through targeting of the CD44-Rho kinase (ROCK) pathway [16]. The present study discovered that silencing of hsa_circ_0023305 promoted miR-218-5p expression but inhibition of miR-218-5p restored the proliferative and invasive capacity of these cells.

Previous studies have identified TRPM7 as a miR-218-5p downstream target, and this was validated by luciferase reporter assay. TRPM7 regulates a nonselective cation channel and enhances cancer metastasis. TRPM7 expression was negatively correlated to E-cadherin expression, but positively correlated to Vimentin, N-cadherin, and Twist expression in ovarian cancer. Depletion of TRPM7 inhibited invasion and migration in OVCAR3 and SKOV3 cells via regulation of phosphoinositide 3-kinsae (PI3K)/protein kinase B (AKT) oncogenic signaling [17]. A study has found that aberrantly expressed circRNAs can mediate cancer progression through regulation of the activity of major signaling cascades, such as the VEGF, WNT/*β*-catenin, MAPK, PI3K/AKT, and Notch signaling pathways, as well as by interfering with signaling crosstalk [18]. Additional studies have demonstrated that TRPM7 is aberrantly expressed in human diseases such as cancer. In cancer cells, TRPM7 functions to promote survival, cell cycle progression, migration, growth, proliferation, invasion, and epithelial-mesenchymal transition (EMT) [19]. In this study, we discovered that silencing of hsa_circ_0023305 decreased TRPM7 expression, but TRPM7 expression was restored in these cells upon inhibition of miR-218-5p. Thus, overexpression of TRPM7 restored the proliferative and invasive ability of cells with silenced expression of hsa_circ_0023305.

## 5. Conclusions

In summary, hsa_circ_0023305 expression was increased in the LSCC patients and the high expression of hsa_circ_0023305 could enhance LSCC cell development and metastasis by upregulating TRPM7. We discovered for the first time that hsa_circ_0023305 binds directly to miR-218-5p and regulated the expression of TRPM7. We supposed that targeting of hsa_circ_0023305 expression may prove to be a novel insight for early diagnosis and genetic therapeutic strategy for LSCC.

## Figures and Tables

**Table 1:** Correlations between hsa_circ_0023305 and clinical characteristics of 30 LSCC patients.

Characteristics	*n*	hsa_circ_0023305 level		*P* value
		High	Low	
Total case	30	22	8	<0.001
Gender				
Male	16	12	4	>0.05
Female	14	10	4	
Age (y)				
≤60	14	10	4	>0.05
>60	16	12	4	
Clinical stage				
I-II	17	11	6	<0.001
III-IV	13	11	2	
Lymph node				
Negative	21	13	8	<0.001
Positive	9	9	0	

## Data Availability

Data produced and analyzed in the study are contained in the paper.
